# 立体定向消融体部放射治疗早期可手术Ⅰ期非小细胞肺癌—距离常规应用有多远?

**DOI:** 10.3779/j.issn.1009-3419.2015.07.02

**Published:** 2015-07-20

**Authors:** 小龙 傅, 旭伟 蔡

**Affiliations:** 200030 上海，上海交通大学附属上海市胸科医院放疗科 Department of Radiation Oncology, Shanghai Chest Hospital, Shanghai Jiao Tong University, 200030 Shanghai, China

一个月之前，美国MD Andeson医院的张玉蛟教授在*The* *Lancet Oncology*发表了对于早期可以手术的非小细胞肺癌(non-small cell lung cancer, NSCLC)立体定向消融体部放射治疗(stereotactic ablative radiotherapy, SABR)与手术切除疗效比较的文章^[1]^。该篇文章一发表，真如一石激起千层浪，引起了广泛争鸣和争论。一个月后，激烈的争鸣和争论似乎也告一段落。平静后，我们再思考这篇文章告诉我们一些什么？

SABR技术诞生在20世纪80年代末和90年代初，简单来说在新的放疗技术平台条件下将放疗时间剂量分割做了很大调整，由过去常规分割放疗(5天/周，1次/天，总剂量60 Gy-70 Gy/30次-35次)调整到整个放疗疗程，在照射5次左右完成，明显提高了放疗剂量强度，大大提高了肿瘤局部控制率。

经过近30年的临床实践，SABR在早期NSCLC的地位是：①不能手术的Ⅰ期NSCLC的标准治疗方案；②因为心肺功能或其他内科疾病造成患者不能耐受标准肺叶切除，是该类患者肿瘤楔形切除外的另一治疗方法选择；③对于可手术的Ⅰ期NSCLC，目前肺叶切除+纵隔淋巴结取样或清扫是其标准治疗。回顾性分析资料虽然显示SABR具有与手术相当的疗效(相似的肿瘤特异性生存率和局控率)，但是尚无临床Ⅲ期研究来回答这一问题，因此一直存在很大争议。

之所以张玉蛟教授的论文引起大家很大关注，最主要是因为这是第一项前瞻性研究比较了SABR与手术疗效的差异性的研究，所涉及的目标患者是可以手术的Ⅰ期NSCLC，挑战的是传统而且非常有效的治疗手段––手术。

针对SABR在早期可以手术Ⅰ期NSCLC的治疗地位，在世界范围内有3项独立的Ⅲ期随机临床研究(STARS、ROSEL和ACOSOG Z4099)。由于患者入组太慢，3项研究均被提前关闭了。张玉蛟教授等收集了前2项研究的资料进行汇集分析(pooled analysis)，纳入2项研究中符合条件的患者，即临床T1-2a( < 4 cm)N0M0，可手术的NSCLC，按1:1随机分配，一组采取传统的手术方式即肺叶切除+纵隔淋巴结清扫或取样，另一组为SABR治疗。主要研究终点为总生存。两项研究共58例患者可以进入分析，其中SABR组31例，手术组27例。结果显示：SABR和手术组中位随访时间分别为40.2个月和35.4个月，死亡患者数分别为1例和6例，3年总生存率分别为95%和79%(HR=0.14, *P*=0.037)，3年无复发生存率分别为86%和80%(HR=0.69, *P*=0.54)。失败表型，SABR组1例局部复发，4例区域淋巴结复发，1例远处转移；手术组1例出现区域淋巴结复发，2例远处转移。毒副反应方面，SABR组3例(10%)患者发生3级治疗相关不良事件，无4级不良事件及治疗相关死亡发生；手术组1例(4%)患者死于手术并发症，12例(44%)患者发生3级-4级治疗相关不良事件。结果提示：SABR可能是可以手术的早期NSCLC另外一个选择，由于样本量小和随访时间短，需要更多临床研究来验证。

这篇文章是第一个来自于前瞻性研究资料，挑战的是Ⅰ期NSCLC传统而且非常有效的治疗手段——手术，且肺癌又是常见社会影响面极其大的恶性肿瘤，本研究初步结果显示对于可手术的Ⅰ期NSCLC，SABR疗效优于手术，毒副反应低于手术，SABR可能是手术外的另一治疗选择。因此，这篇文章引起学界特别是外科医生的高度关注也是可以理解的。

该研究的优势：所收集的患者是来自于前瞻性研究入组患者，影响患者预后的临床因素诊断和分期检查等均做了控制，特别是治疗不确定性因素如放疗技术环节上的放疗剂量，正常组织器官安全剂量限制，质量控制和保证等均进行了有效控制，提高了手术和SABR治疗技术实施的一致性。

该研究存在的劣势：①病例数太少。即使集合了2项临床研究，也仅有可怜的58例患者，只要有1例治疗相关死亡就将对总生存产生较大影响。而集合了2项研究的数据，虽然这2项研究患者入组标准相似，但是患者基线资料仍很难进行细致比较，容易出现选择偏倚。因此很多外科医生认为本研究中Ⅰ期NSCLC术后3年生存率为79%不能代表外科治疗该期别患者的总体水平，而SABR组的3年生存率高达95%也好于历史对照，这些可能均与样本量小患者的选择偏倚有关；②随访时间太短，只报道了3年总生存率。对于Ⅰ期NSCLC，5年生存率是起码的评判标准，我们还需要再等待进一步的随访结果；③入组的27例患者多数采取的是传统开胸治疗方法，只有少数采取微创手术方式，这至少影响到手术组副反应发生率高低的观察；④毕竟是两项研究的pooled analysis，因此从课题设计和统计学分析看，所得到统计学结论具有一些偶然性，可信程度相对低。

这篇文章能告诉我们什么？①过去对于SABR治疗早期特别是可以手术的早期NSCLC的认同程度低。就像这3项研究一样为何会入组缓慢而终止？我们看下STARS研究，筛选失败的患者中除了不符合入组标准的，其中有66例是因为对治疗方式有主观选择性而不能入组，这66例中54例患者选择了手术，仅有9例患者选择SABR，还有3例患者选择放弃治疗；②SABR治疗副反应低(无治疗相关性死亡，≥Ⅲ级治疗相关性损伤在10%左右)，SABR用于不可手术或拒绝手术Ⅰ期NSCLC有很多年，也积累了大量患者治疗的临床经验^[2]^，本研究所观察到SABR治疗早期可以手术的NSCLC副反应与以往所报道的不能耐受手术或拒绝手术早期患者SABR治疗反应一致，再次验证了SABR治疗安全性高、副作用小；③与以往的回顾性分析^[3, 4]^比较，在早期可以手术Ⅰ期NSCLC中，我们增加了一条前瞻性研究资料证实SABR疗效仍然是很好的。

这篇文章不能说明什么？在早期可以手术的Ⅰ期NSCLC，SABR与手术切除之间是否存在疗效差异？特别是长期生存疗效方面是否存在差异性？不能说明某种优于另一种治疗方式。

这篇文章最主要贡献在于：现在争论SABR与手术之间疗效水平高低已经没有多大意义，可以预见在不久的将来将有更多和更大样本临床Ⅲ期研究来关注这个问题。但问题的关键是这样的临床研究能否进行下去？张玉蛟教授的文章提高了社会(包括医生、患者及家属)对SABR治疗的认识水平，让更多人关心此技术的临床价值，有更多人愿意组织和参与类似临床研究，增加了患者对SABR治疗依从性，能明显推进SABR治疗临床价值的相关临床研究深入广泛开展^[5]^。我觉得这才是这篇文章的最大贡献，这也是该研究尽管有许多缺陷，但仍能发表在世界顶级肿瘤学术期刊上的主要原因。

随着这篇文章所引发的Ⅰ期可以手术NSCLC如何进行临床治疗争论的硝烟散去，留下来更多的是我们需要冷静的思考。我们需要客观、科学和平等地看待已有的临床证据，思考是否已经具备了开展让更多治疗手段参与可手术Ⅰ期NSCLC治疗临床前瞻性研究的基础。我们需要更多、更大样本临床前瞻性研究。我们相信在不远的将来，临床上将为Ⅰ期NSCLC治疗提供更多证据和更多选择。

## References

[1] Chang JY, Senan S, Paul MA (2015). Stereotactic ablative radiotherapy versus lobectomy for operable stage Ⅰ non-small-cell lung cancer: a pooled analysis of two randomised trials. Lancet Oncol.

[2] Chi A, Liao Z, Nguyen NP (2010). Systemic review of the patterns of failure following stereotactic body radiation therapy in early-stage non-small-cell lung cancer: clinical implications. Radiother Oncol.

[3] Onishi H, Shirato H, Nagata Y (2011). Stereotactic body radiotherapy (SBRT) for operable stage Ⅰ non-small-cell lung cancer: can SBRT be comparable to surgery?. Int J Radiat Oncol Biol Phys.

[4] Lagerwaard FJ, Verstegen NE, Haasbeek CJ (2012). Outcomes of stereotactic ablative radiotherapy in patients with potentially operable stage Ⅰ non-small cell lung cancer. Int J Radiat Oncol Biol Phys.

[5] Treasure T, Rintoul RC, Macbeth F (2015). SABR in early operable lung cancer: time for evidence. Lancet Oncol.

